# Supplementary material for: Differences in GIP Receptor Expression by Feeding Status in the Mouse Brain
Source: Int J Mol Sci. 2025 Jan 28;26(3):1142. doi: 10.3390/ijms26031142 (PMC11818402; doi:10.3390/ijms26031142)
Supplement: Supplementary file 1 [file ijms-26-01142-s001.zip › ijms-3414714-supplementary.pdf]

International Journal of
Molecular Sciences

MDPI

**Table S1.** Primer sequences used for real time PCR analysis.

| Gene | Forward | Reverse |
|---|---|---|
| *Gipr* | 5′- CTGCCTGCCGCACGGCCCAGAT-3′ | 5′- GCGAGCCAGCCTCAGCCGGTAA-3′ |
| *Lepr* | 5′- GATTTCTTGGGACAGCCAAA-3′ | 5′- TCCAGACTCCTGAACCATCC-3′ |
| *Pomc* | 5′- CTAAGAGAGGCCACTGAACA-3′ | 5′- TCTATGGAGGTCTGAAGCAG-3′ |
| *Cart* | 5′- TGCTGGGATTAAAGGCGTGT-3′ | 5′-TCTCTGAGGGGAACGCAAAC-3′ |
| *Npy* | 5′- GGTGGATCTCTTCTCTCACA-3′ | 5′- CAGAGCGGAGTAGTATCTGG-3′ |
| *Agrp* | 5′-TAGGTGCGACTACAGAGGTT-3′ | 5′- GAGGTGCTAGATCCACAGAA-3′ |

*Gipr*, gastric inhibitory polypeptide receptor; *Lepr*, leptin receptor; *Pomc*, proopiomelanocortin; *Cart*, cocaine and amphetamine regulat-ed transcript; *Npy*, neuropeptide Y; *Agrp*, agouti-related peptide.

 https://doi.org/10.3390/ijms26031142
